# Polychromatic Flow Cytometric Analysis of Stromal Vascular Fraction from Lipoaspirate and Microfragmented Counterparts Reveals Sex-Related Immunophenotype Differences

**DOI:** 10.3390/genes12121999

**Published:** 2021-12-16

**Authors:** Lucija Zenic, Denis Polancec, Damir Hudetz, Zeljko Jelec, Eduard Rod, Dinko Vidovic, Mario Staresinic, Srecko Sabalic, Trpimir Vrdoljak, Tadija Petrovic, Fabijan Cukelj, Vilim Molnar, Martin Cemerin, Vid Matisic, Petar Brlek, Zrinka Djukic Koroljevic, Igor Boric, Gordan Lauc, Dragan Primorac

**Affiliations:** 1 Department for Translational Medicine, Srebrnjak Children’s Hospital, 10000 Zagreb, Croatia; dpolancec@bolnica-srebrnjak.hr; 2 St. Catherine Specialty Hospital, 10000 Zagreb, Croatia; ortohud@gmail.com (D.H.); zeljko.jelec@svkatarina.hr (Z.J.); eduard.rod@svkatarina.hr (E.R.); dinko.vidovic@gmail.com (D.V.); trpimir.vrdoljak@svkatarina.hr (T.V.); vilim.molnar@svkatarina.hr (V.M.); martincemerin@gmail.com (M.C.); vid.matisic@svkatarina.hr (V.M.); petar.brlek@svkatarina.hr (P.B.); zrinka.djukickoroljevic@svkatarina.hr (Z.D.K.); igor.boric@svkatarina.hr (I.B.); draganprimorac2@gmail.com (D.P.); 3 Clinical Hospital Sveti Duh, 10000 Zagreb, Croatia; 4 School of Medicine, Josip Juraj Strossmayer University of Osijek, 31000 Osijek, Croatia; 5 Department of Nursing, University North, 48000 Varaždin, Croatia; 6 Clinic for Traumatology, University Hospital Sestre Milosrdnice, Draškovićeva 19, 10000 Zagreb, Croatia; ssabalic@gmail.com (S.S.); tadijap@gmail.com (T.P.); fabijan.cukelj@svkatarina.hr (F.C.); 7 School of Dental Medicine, University of Zagreb, 10 000 Zagreb, Croatia; 8 Department of Traumatology, Medical University Merkur Hospital, 10000 Zagreb, Croatia; mstaresinic@yahoo.com; 9 Medical School, University of Zagreb, 10000 Zagreb, Croatia; 10 Medical School, University of Split, 21000 Split, Croatia; 11 Medical School, University of Rijeka, 51000 Rijeka, Croatia; 12 Medical School, University of Mostar, 88000 Mostar, Bosnia and Herzegovina; 13 Department of Health Studies, University of Split, 21000 Split, Croatia; 14 Genos Glycoscience Research Laboratory, 10000 Zagreb, Croatia; glauc@genos.hr; 15 Faculty of Pharmacy and Biochemistry, University of Zagreb, 10000 Zagreb, Croatia; 16 Eberly College of Science, The Pennsylvania State University, University Park, State College, PA 16802, USA; 17 The Henry C. Lee College of Criminal Justice and Forensic Sciences, University of New Haven, West Haven, CT 06516, USA; 18 Faculty of Dental Medicine and Health, Josip Juraj Strossmayer University of Osijek, 31000 Osijek, Croatia; 19 Medical School REGIOMED, 96450 Coburg, Germany

**Keywords:** osteoarthritis, mesenchymal stem/stromal cells, stromal vascular fraction, lipoaspirate, microfragmentation, immunophenotyping, endothelial progenitors, pericytes, supra-adventitial-adipose stromal cells

## Abstract

Mesenchymal stem/stromal cells or medicinal signaling cells (MSC)-based therapy holds promise as a beneficial strategy for treating knee OA (osteoarthritis), but there is no standardized protocols nor mechanistic understanding. In order to gain a better insight into the human MSC from adipose tissue applied for autologous OA treatment, we performed extensive comparative immunophenotyping of the stromal vascular fraction from lipoaspirate or microfragmented lipoaspirates by polychromatic flow cytometry and investigated the cellular components considered responsible for cartilage regeneration. We found an enrichment of the regenerative cellular niche of the clinically applied microfragmented stromal vascular fraction. Sex-related differences were observed in the MSC marker expression and the ratio of the progenitor cells from fresh lipoaspirate, which, in female patients, contained a higher expression of CD90 on the three progenitor cell types including pericytes, a higher expression of CD105 and CD146 on CD31^high^CD34^high^ endothelial progenitors as well as of CD73 on supra-adventitialadipose stromal cells. Some of these MSC-expression differences were present after microfragmentation and indicated a differential phenotype pattern of the applied MSC mixture in female and male patients. Our results provide a better insight into the heterogeneity of the adipose MSC subpopulations serving as OA therapeutics, with an emphasis on interesting differences between women and men.

## 1. Introduction

Osteoarthritis (OA) is the most common musculoskeletal progressive disease and the most common cause of disability in old age worldwide. Due to the lack of disease biomarkers, effective targets and complex pathogenesis, OA treatment remains a great challenge [1]. Current therapies help manage the condition but do not provoke regeneration of degenerated tissues. Therapeutic options in regenerative medicine, such as the application of platelet-enriched plasma and mesenchymal stem/stromal cells, i.e., medicinal signaling cells (MSC), are the latest promising methods to be investigated. Clinical application of the stromal vascular fraction from adipose tissue harboring MSC has shown a significant effect in reducing pain in patients with knee OA and also significant shifts in the quality of hyaline cartilage [2,3,4]. The efficacy of these MSC in tissue repair has been shown by several studies of knee OA treatment and application of even a low-dose MSC from adipose tissue resulting in significant pain relief and functional improvement [5,6,7]. Preferential features of MSC attributed to their therapeutic effects are the ability to stimulate cartilage formation, vascularization, anti-inflammation and immunomodulation [8].

However, the guidelines of professional societies do not recommend the use of MSC for the treatment of OA, mainly due to insufficiently standardized protocols for collecting and isolating cells from autologous tissue and application into a diseased joint [9]. This reluctance also stems from the incomplete understanding of MSC heterogeneity as well as analytical approaches to cell characterization. Besides MSC, a variety of cell populations constitute the stromal vascular fraction, including endothelial progenitors and pericytes. Recent reports have emphasized the importance of their interactions and challenge the view of their reciprocal in vivo origin [10,11,12,13]. There is a substantial discrepancy in the reports of the surface marker expression on MSC freshly isolated from adipose tissue, e.g., CD105 [14,15] and CD146 [15,16,17]. Therefore, both a more thorough translational research approach and a uniform methodology of MSC treatment are called for.

To this end, the aim of this study was to immunophenotype cells from the stromal vascular fraction obtained from lipoaspirate or processed lipoaspirate applied for OA treatment using polychromatic flow cytometry to investigate the cellular components considered responsible for cartilage regeneration. We employed a comprehensive panel analysis using not only a percentage-positive approach to define the heterogeneous cell types and ratios but also the fluorescence intensity correlating to receptor quantity, enabling the cell subpopulation characterization and sex-related changes in marker expression.

## 2. Results

### 2.1. Immunophenotyping of Stromal Vascular Fraction by Polychromatic Flow Cytometry

**Figure 1:**
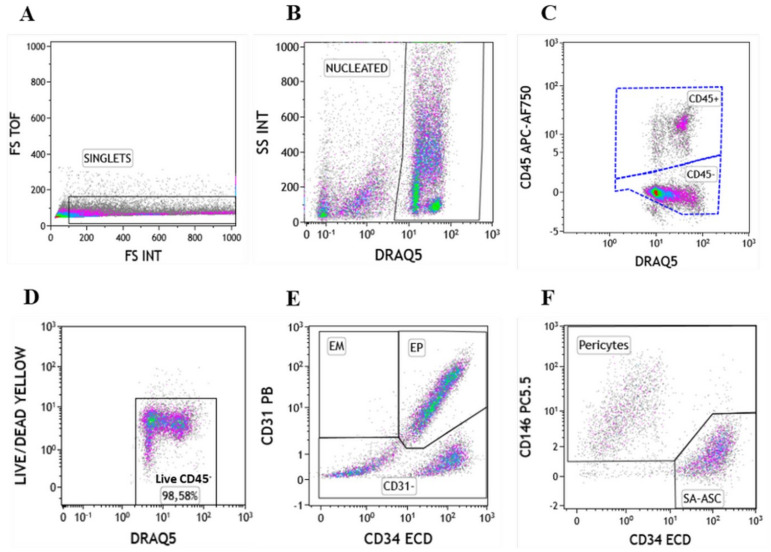
The gating procedure for the polychromatic flow cytometry analysis of heterogeneous cell content in the stromal vascular fraction from lipoaspirate, microfragmented lipoaspirate and concentrated stromal vascular fraction samples. Singlet events based on forward scatter (FS) intensity (INT) and FS time of flight (TOF) (**A**) and nucleated cell events selected by the DNA–binding DRAQ5 dye–positivity and side scatter (SC) INT (**B**) were used to analyze the CD45^+^ and CD45^−^ cell populations (**C**), the viability of which was determined based on the Live/Dead Yellow staining (**D**). Nucleated live CD45^−^ cells were phenotyped using the CD31 and CD34 lineage markers, such as CD31^+^CD34^−^ endothelial mature (EM), CD31^+^CD34^+^ endothelial progenitor (EP) and CD31^−^ non–endothelial population (**E**), which was in combination with the CD146 marker further phenotyped as pericytes and supra–adventitial–adipose stromal cells (SA-ASC) (**F**).

Based on our previous study [18], we phenotypically characterized cell subpopulations of the stromal vascular fraction from lipoaspirate, microfragmented lipoaspirate and concentrated stromal vascular fraction samples, named LA, MLA and SVF, respectively. The gating procedure, targeting single nucleated CD45^+^ and CD45^−^ populations, is shown in Figure 1A–C. From here, we analyzed the CD45^−^ subpopulation for viability that averaged greater than 98%, and only live cells were included in further analysis (Figure 1D). Lineage and cell adhesion markers were used to gain insight into the non-leukocyte subpopulations (Figure 1E,F).

Using this gating strategy, we determined the phenotypes of the CD45^−^ subpopulation in LA, MLA and SVF samples, including the following: CD31^+^CD34^+^CD73^±^CD90^±^CD105^±^CD146^±^ endothelial progenitors (EP), CD31^+^CD34^−^CD73^±^CD90^±^CD105^−^CD146^±^ endothelial mature (EM), CD31^−^CD34^−^CD73^±^CD90^+^CD105^−^CD146^+^ pericytes, CD31^−^CD34^+^CD73^±^CD90^+^CD105^−^CD146^+^ transitional pericytes and CD31^−^CD34^+^CD73^high^CD90^+^CD105^−^CD146^−^ supra-adventitial-adipose stromal cells (SA-ASC) (Table 1).

### 2.2. Regenerative Cell Enrichment of Stromal Vascular Fraction

**Figure 2:**
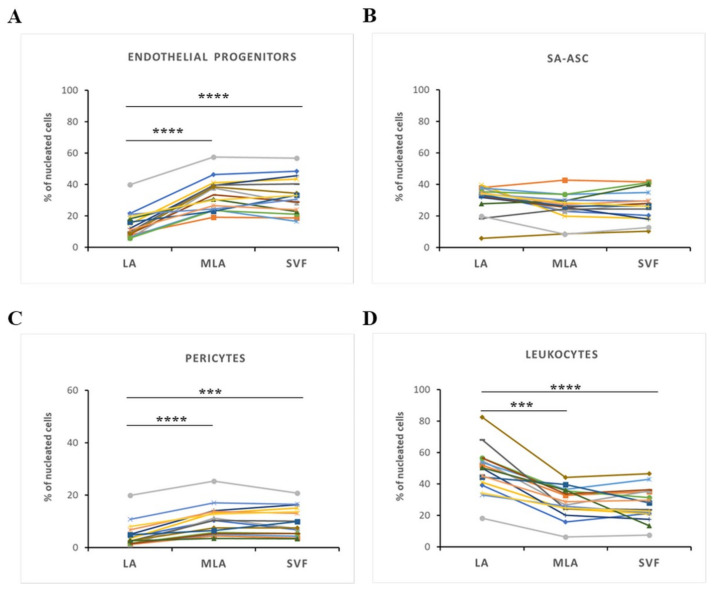
The main cell populations in stromal vascular fraction obtained from LA, MLA and SVF samples. The difference in the cell content between LA, MLA and SVF samples is shown as a percentage of nucleated cells for endothelial progenitors (**A**), SA-ASC (**B**), pericytes (**C**) and leukocytes (**D**) for each patient. Statistical analysis was performed using ordinary one-way ANOVA with Dunn’s multiple comparisons test (**A**,**D**) or Friedman’s multiple comparisons test (**B**,**C**). *p*-values: (***) *p* < 0.001, (****) *p* < 0.0001; *n* = 16.

In order to determine the cellular proportions of heterogeneous stromal content obtained by lipoaspiration and microfragmented lipoaspirate for clinical application, we observed four main cell subpopulations in the relative amounts of phenotypic markers expressed as a percentage of live nucleated cells (Figure 2). The percentage of EP and pericytes was significantly higher in MLA and SVF compared to LA (Figure 2A,C), while the percentage of leukocytes was significantly lower (Figure 2D). Interestingly, the percentage of SA-ASC showed a donor-dependent variability and did not change significantly between the sample groups (Figure 2B). The increase in pericytes and EP, as well as the decrease in leukocytes, did not differ between MLA and SVF samples, i.e., no difference was seen between MLA samples obtained after one centrifugation and SVF samples obtained after two centrifugations using the Arthrex ACP^®^ Double-Syringe System (Figure 2).

### 2.3. Sex-Related Differences in the Stromal Progenitor Cells

**Figure 3:**
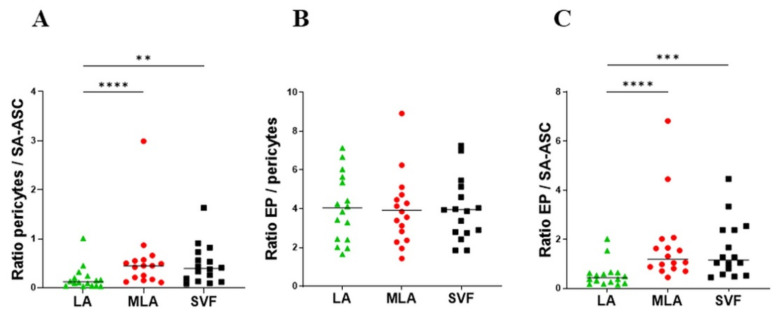
Differences in the ratios of the progenitor cells in the stromal vascular fraction from LA, MLA and SVF samples. The pericyte/SA-ASC ratio (**A**), the EP/pericyte ratio (**B**) and the EP/SA-ASC ratio (**C**) were calculated from the quantitative data shown in Figure 2 (percentage of nucleated cells for each cell population). The data are expressed as symbols representing each patient with the group median (**A**,**C**) or mean (**B**). Statistical analysis was performed using the Friedman multiple comparisons test (**A**,**C**) or ordinary one-way ANOVA with Tukey’s multiple comparison test (**B**). *p*-values: (**) *p* < 0.01; (***) *p* < 0.001, (****) *p* < 0.0001; *n* = 16.

**Figure 4:**
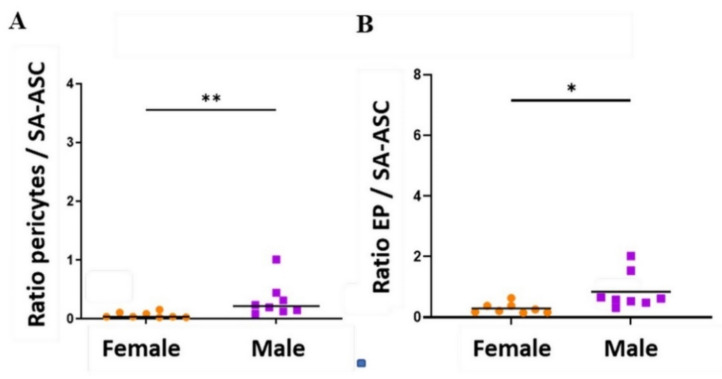
Differences between female and male patients in the progenitor cell ratios in the stromal vascular fraction from LA samples. The pericyte/SA-ASC ratio (**A**) and the EP/SA-ASC ratio (**B**) were calculated from the quantitative data shown in Figure 2 (percentage of nucleated cells for each cell population). The data are expressed as symbols representing each female patient (*n* = 8) or male patient (*n* = 8) with the group median (**A**) or mean (**B**). Statistical analysis was performed using the Mann–Whitney test (**A**) or unpaired t-test (**B**). *p*-values: (*) *p* < 0.05 (**) *p* < 0.01.

In line with the enrichment of the regenerative cell potential, we looked at the ratios of the progenitor cells before and after microfragmentation (Figure 3). The pericytes/SA-ASC and EP/SA-ASC ratios (Figure 3A,C), but not the EP/pericytes ratio (Figure 3B), were significantly higher in MLA and SVF samples compared to LA samples. The same ratios showed a statistical difference when we investigated the sex-related basis and compared the samples from female and male patients. However, statistically higher ratios in men were only seen in LA (Figure 4) but not in MLA and SVF samples (Supplementary Figure S1).

### 2.4. Sex-Related Heterogeneity of the Precursor Subpopulations

**Figure 5:**
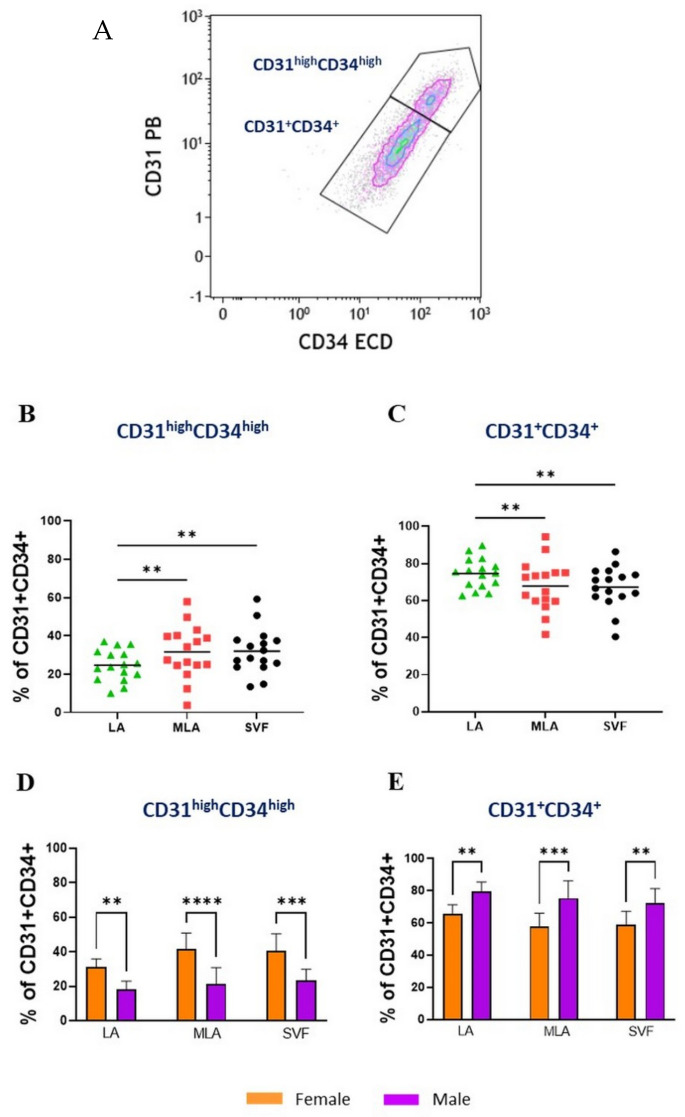
Phenotypic analysis of the EP cell subpopulations using CD31 and CD34 marker expression. The gating of EP based on the CD34 and CD31 marker (shown in Figure 1E) served for a further selection and discrimination of CD31^+^CD34^+^ EP and CD31^high^CD34^high^ EP subpopulations (**A**). Differences in the proportion of CD31^high^CD34^high^ EP (**B**) and CD31^+^CD34^+^ EP (**C**) (expressed as a percentage of CD31^+^CD34^+^ EP) between LA, MLA and SVF samples. The data are expressed as symbols representing each patient with the group mean. Statistical analysis was performed using one–way ANOVA with Tukey’s multiple comparison test. *p*-values: (**) *p* < 0.01; *n* = 16. Differences in the proportion of CD31^high^CD34^high^ EP (**D**) and CD31^+^CD34^+^ EP (**E**) between female and male patients in LA, MLA and SVF samples. The data are expressed as bars with SD of 8 patients. Statistical analysis was performed using ordinary one–way ANOVA with Sidak’s multiple comparison test. *p*-values: (**) *p* < 0.01; (***) *p* < 0.001; (****) *p* < 0.0001; *n* = 8.

Focusing further on the subtle differences in the lineage marker expression, we noticed a differential expression of the CD31 and CD34 markers reflecting two subpopulations of EP cells: the more abundant CD31^+^CD34^+^ EP and the less abundant CD31^high^CD34^high^ EP (Figure 5A). The proportion of the CD31^high^CD34^high^ EP (expressed as the percentage of EP) was significantly higher (Figure 5B), while the proportion of CD31^+^CD34^+^ EP was statistically lower (Figure 5C) in MLA and SVF compared to LA. Furthermore, the level of the CD31 and CD34 expression differed based on sex, and in female patients, the proportion of CD31^high^CD34^high^ EP was statistically higher (Figure 5D), while the proportion of CD31^+^CD34^+^ EP was statistically lower compared to male patients in all LA, MLA and SVF samples (Figure 5E).

**Figure 6:**
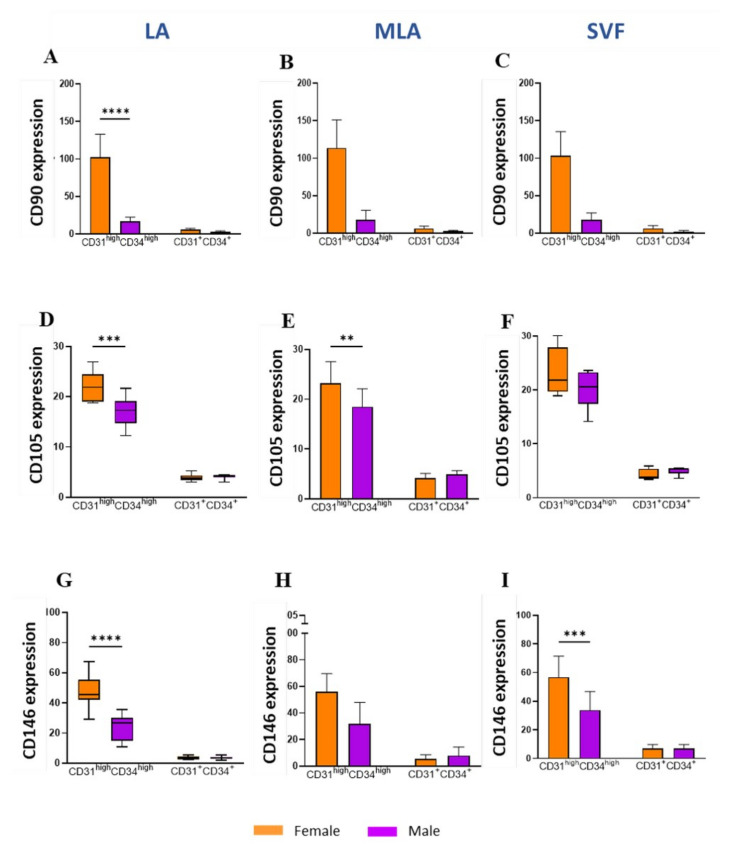
Expression of the mesenchymal stem/stromal cell-characteristic markers in EP subpopulations. Differences between female and male patients in the expression of CD90 (**A**–**C**), CD105 (**D**–**F)** and CD146 (**G**–**I**) markers on CD31^high^CD34^high^ EP and CD31^+^CD34^+^ EP from LA (left panels), MLA (middle panels) and SVF samples (right panels). The geometric mean fluorescence intensity (geo MFI) data are expressed as bars with SD (**A**–**C**,**E**,**H**,**I**) or box and whiskers (**D**,**F**,**G**) of 8 patients. Statistical analysis was performed using ordinary one–way ANOVA with Sidak’s multiple comparison test (**A**–**C**,**E**,**H**,**I**) or Kruskal–Wallis with Dunn’s multiple comparisons test (**D**,**F**,**G**). *p*-values: (**) *p* < 0.01; (***) *p* < 0.001; (****) *p* < 0.0001.

When we explored the MSC marker expression (Figure 6), we found significantly higher expression of CD90 (Figure 6A), CD105 (Figure 6D) and CD146 (Figure 6G) on CD31^high^CD34^high^ EP in LA from female patients compared to their male counterparts. The same CD31^high^CD34^high^ EP subpopulation showed a significantly higher expression of CD105 in MLA samples (Figure 6E) and CD146 in SVF samples (Figure 6I) from female patients compared to male patients.

**Figure 7:**
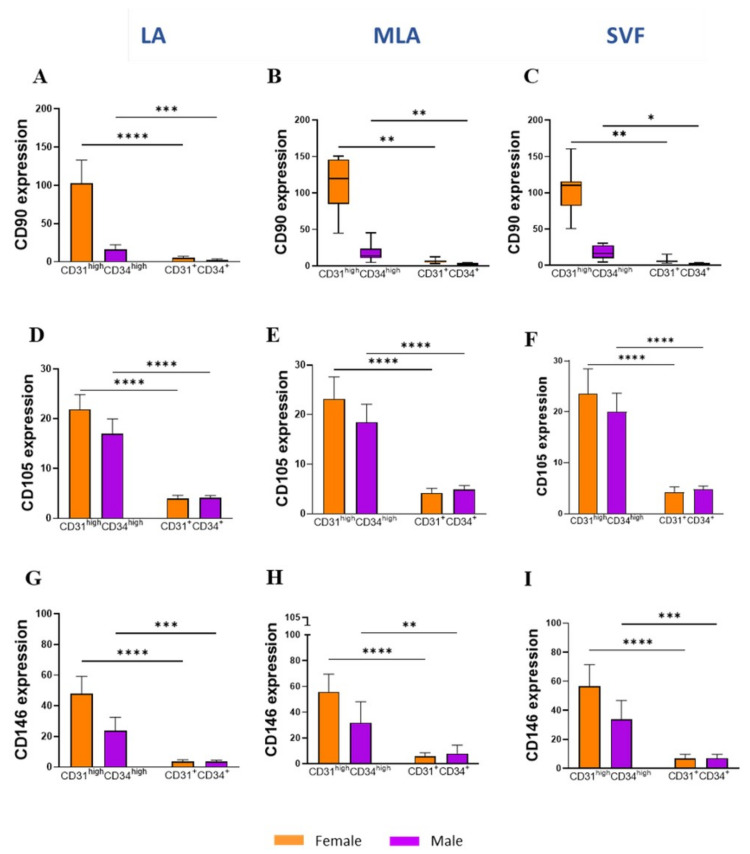
Expression of the mesenchymal stem/stromal cell–characteristic markers in EP subpopulations. Differences in the expression of CD90 (**A**–**C**), CD105 (**D**–**F**) and CD146 (**G**–**I**) markers between CD31^high^CD34^high^ EP and CD31^+^CD34^+^ EP from LA (left panels), MLA (middle panels) and SVF samples (right panels) in female and male patients. The geometric mean fluorescence intensity (geo MFI) data are expressed as bars with SD (**A**,**D**–**I**) or box and whiskers (**B**,**C**) of 8 patients. Statistical analysis was performed using ordinary one–way ANOVA with Sidak’s multiple comparison test (**A**,**D**–**I**) or Friedman multiple comparisons test (**B**,**C**). *p*-values: (*) *p* < 0.05; (**) *p* < 0.01; (***) *p* < 0.001; (****) *p* < 0.0001.

When the same data were compared for CD31^+^CD34^+^ EP and CD31^high^CD34^high^ EP within each sex, we found statistically higher expression of CD90 (Figure 7A–C), CD105 (Figure 7D–F) and CD146 (Figure 7G–I) markers on CD31^high^CD34^high^ EP in both female and male patients compared to their expression on CD31^+^CD34^+^ from all LA, MLA and SVF samples.

**Figure 8:**
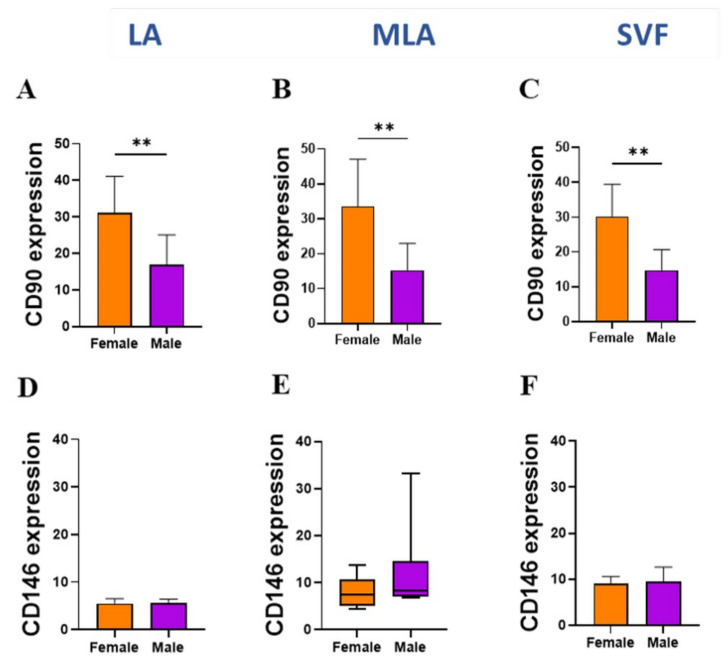
Expression of cell surface markers in pericytes. Differences between female and male patients in the expression of CD90 (**A**–**C**) and CD146 (**D**–**F**) markers from LA, MLA and SVF samples. The geometric mean fluorescence intensity (geo MFI) data are expressed as bars with SD of 8 patients for all graphs except one box and whiskers (**E**). Statistical analysis was performed using an unpaired t–test, except for the Mann–Whitney test in E. *p*-values (**) *p* < 0.01.

**Figure 9:**
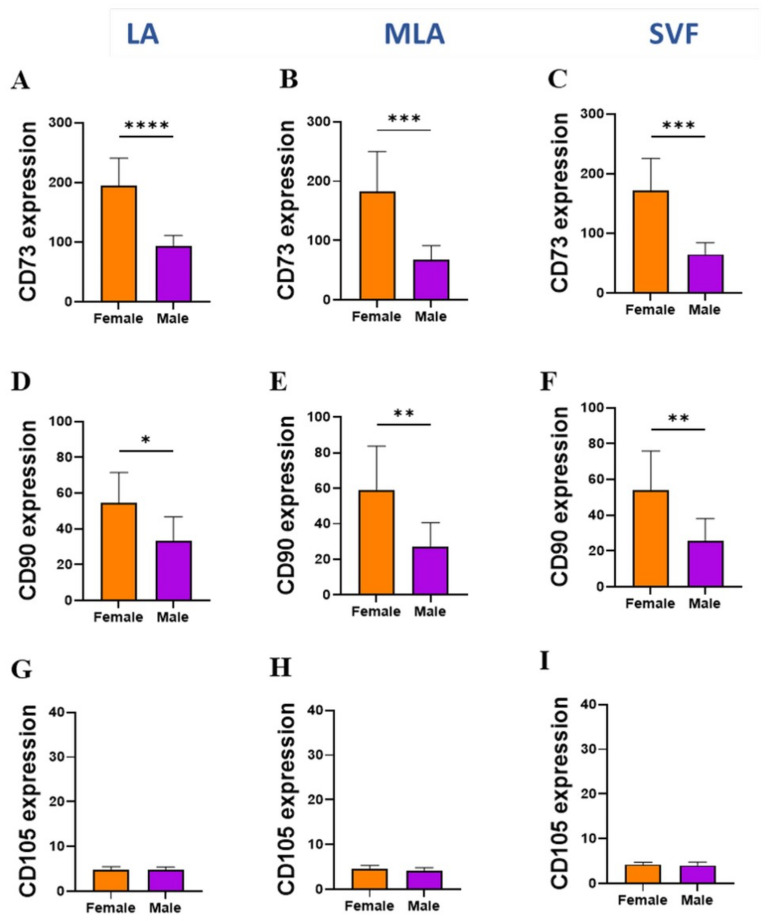
Expression of cell surface markers in SA–ASC. Differences between female and male patients in the expression of CD73 (**A**–**C**), CD90 (**D**–**F**) and CD105 (**G**–**I**) markers on SA–ASC from LA, MLA and SVF samples. The geometric mean fluorescence intensity (geo MFI) data are expressed as bars with SD of 8 patients. Statistical analysis was performed using unpaired *t*-test. *p*-values: (*) *p* < 0.05; (**) *p* < 0.01; (***) *p* < 0.001; (****) *p* < 0.0001.

When we analyzed the MSC markers and CD146 adhesion molecule expression in other progenitor populations (Figure 8 and Figure 9), female patients showed a significantly higher expression of CD90 on pericytes (Figure 8A–C) as well as CD73 and CD90 on SA-ASC from all LA, MLA and SVF samples (Figure 9A–F).

## 3. Discussion

Although we are aware of the terminology issue accompanying the MSC research and clinical application [19,20], in this paper, we have primarily defined the heterogeneous cell content of adipose-derived SVF applied for the treatment of OA. We found an increase in EP and pericytes together with a decrease in leukocytes in the course of SVF microfragmentation and concentration. Since pericytes are considered the in vivo progenitors of MSC, and given the presumed role of EP in tissue regeneration, our results showed a marked enrichment of the regenerative cellular niche in the autologous conditioned adipose tissue technology applied in orthopedic therapy. In all the comparative analyses throughout the study, we observed the same results with MLA or SVF samples and suggest that the method of lipoaspirate microfragmentation involving one centrifugation step using the Arthrex ACP^®^ Double-Syringe System is equally effective in the enrichment of the regenerative cell compartment as the method of concentrating SVF involving two centrifugation steps using the same system (Figure 2).

In our previous study detailing the immunophenotyping profile of SVF obtained by a similar microfragmentation technology [18], which provoked an improved clinical and functional outcome in 85% of patients with late-stage knee OA [3], we already witnessed a high enrichment of EP cells and emphasized their implication in tissue regeneration. We believe that the demonstrated crosstalk between MSC and EP [21,22,23] might form an axis for the action of MSC as medicinal signaling cells [19]. As with our previous experience [18,24], we here looked for the sex-related variations and found that ratios in the three progenitor cells reflected differences between women and men, which was seen in lipoaspirate samples (LA) but not in the microfragmented (MLA, SVF) samples (Figure 3 and Figure 4).

Given the high percentage of EP applied intra-articularly that we here observed (Figure 2), we aimed to examine subtle expression variations in those cells functionally involved in angiogenesis and tissue repair. Based on the high expression level of CD31 and CD34 (Figure 5A), we found that the CD31^high^CD34^high^ EP phenotype was enriched, and the CD31^+^CD34^+^ EP phenotype was reduced in the course of microfragmentation (Figure 5B,C). Since CD31 is the earliest endothelial marker and CD31^high^ endothelial precursors from blood have been shown to differentiate more efficiently into endothelial cells [25], a higher percentage of CD31^high^CD34^high^ EP in our female patients (Figure 5D,E) might indicate the presence of a more differentiated EP phenotype in women. In a further female–male disparity, we found a higher expression of CD90, CD105 and CD146 in cells from lipoaspirate of female patients, which suggests a higher expression of MSC-associated markers in women (Figure 6). Of the two phenotypes, CD31^high^CD34^high^ EP exhibited a higher expression of the MSC-associated markers in both female and male patients in all samples alike (Figure 7). The two perivascular cell subsets, pericytes and SA-ASC, in female patients, showed a higher expression of CD90 and also CD73 in the latter, both before and after microfragmentation (Figure 8 and Figure 9).

Although renowned as MSC signature markers, the expression of CD73, CD90, CD105 and CD146 are far from uniform and their function in MSC biology is still little understood. Special attention needs to be paid to the sparse literature data from human native as opposed to the more common cultured MSC, as the expression of signature and functional markers appear to be donor-dependent or change during the ex vivo-manipulation of MSC [16,26,27].

However, CD73 expression on MSC has been associated with their anti-inflammatory effects and reparative properties [28], and on unpassaged subcutaneous adipose tissue MSC, the expression of CD73 gave a higher proliferative capacity and increased stem cell marker expression [29].

CD90 has been proposed as a critical target for regulating adipose-derived stem cells based on its higher expression on subcutaneous adipose-derived stem cells in obese patients compared to their visceral counterparts, which, in mice studies, led to a higher proliferation and capacity for adipogenic differentiation [30]. On the other hand, CD90 knockdown in human adipose MSC-facilitated osteogenic and adipogenic differentiation, implicating the role of CD90 in maintaining an undifferentiated state of MSC [31]. In a recent study, CD90, together with CD73 expression, was not altered on cultured ex vivo adipose-derived MSC compared to uncultured adipose MSC; yet it was accompanied by a reduced expression of genes associated with proliferation and differentiation [32].

The expression of CD105, a marker of neovascularization that is predominantly expressed on vascular system cells and involved in endothelial cell survival [33], has been considerably dependent on MSC culture conditions and time [34]. Although some papers have reported the presence of CD105 on freshly isolated SVF cells from adipose tissue [35,36,37], we found a substantial expression of CD105 only on EP cells but not on SA-ASC and pericytes, which showed weak or absent CD105 expression, respectively (Figure 9G–I; not shown in Figure 8); the same held true for microfragmented samples (MLA, SVF). Similar results were obtained in our previous paper [18] and by another study where the CD105 expression was noticeably low on untouched adipose tissue-derived stromal cells of the CD34^+^CD73^+^CD90^+^ phenotype, corresponding to our SA-ASC [32].

The CD146 transmembrane immunoglobulin involved in angiogenesis can be expressed in endothelial cells in different isoforms with distinct functions in vessel regeneration, and an overexpression of certain isoforms in EP increases proangiogenic potential [38]. Within a bone marrow MSC population, a higher expression of CD146 on certain subpopulations is, amongst other effects, associated with greater immunomodulatory and anti-inflammatory protein production [39]**.**

Overall, we noticed that lipoaspirate of female patients contained a higher expression of CD90 on all three progenitor cell types, a difference that was in the two perivascular cell subsets kept after microfragmentation and concentration. Adipose samples from female patients furthermore contained a higher expression of CD105 and CD146 on CD31^high^CD34^high^ EP, as well as CD73 on SA-ASC, the latter difference also being maintained after microfragmentation.

It has been demonstrated that adipose tissue-derived stem cells from different adipose depots, which differ in women and men, exhibit a vast heterogeneity [40]. Differences in adipose-derived stem cells are seen as a more rapid proliferation or a higher osteogenic potential in men than women [41,42]. Transcriptome analysis in female and male adipose-derived stem cells reflects a diverse expression of genes related to inflammation, adipogenic and neurogenic differentiation and cell communication [43]. Specifically, an osteogenic marker SPP1 has been shown to be under-expressed in male adipose-derived stem cells. The obvious discrepancies are partly being explained by the amount of protein encoded by the genes involved in osteogenesis, which emphasizes the importance of a quantitative aspect of protein production. In animal models, female bone marrow-derived MSC secrete more anti-inflammatory and pro-angiogenic factors, which consequently exert a greater therapeutic efficacy in reducing inflammation and vascular remodeling or promoting angiogenesis and alveolarization [44]. At this point, we can only speculate on the functional repercussion of the different MSC marker expression and progenitor ratios in women and men that we observed. However, if aiming at a personalized approach, sexual dimorphism needs to be taken into account in regenerative medicine when improving MSC as anti-inflammatory, immunomodulatory, immunotolerant and pro-angiogenic therapeutics [45,46].

## 4. Materials and Methods

### 4.1. Patients

The study involved sixteen patients with OA (eight females and eight males, aged 33–66) receiving intra-articular knee injection of autologous adipose-derived stromal vascular fraction together with platelet-rich plasma in the St. Catherine Specialty Hospital (Zabok, Croatia). The patient inclusion and exclusion criteria were as follows:

Inclusion criteria:

- patients with knee osteoarthritis;
- patients older than 18 years and younger than 75 years.

Exclusion criteria:

- patients with malignant disease;
- patients with systemic inflammatory diseases (e.g., rheumatoid arthritis);
- patients with grade IV chondromalacia according to the ICRS classification;
- patients with mechanical axis deviation (valgus/varus) of the lower extremities greater than 5 degrees;
- patients with an unstable knee;
- patients with acute meniscal lesions or injuries of other knee structures as the main cause of pain and other symptoms;
- patients with a history of knee surgery;
- patients with mental illness (patients in whom cooperation cannot be expected during the project);
- patients who are found to be unable to respond to follow-up examinations.

### 4.2. Lipoaspiration and Sample Collection

The selected 16 patients (8 women, 8 men) who met the conditions to be included in the study were prepared for the procedure of abdominal subcutaneous adipose tissue aspiration (lipoaspiration) and application of adipose-derived stromal vascular fraction (Arthrex GmbH, Munich, Germany) in combination with platelet-rich plasma from Angel^®^ System (Arthrex GmbH, Munich, Germany).

The lipoaspiration procedure was performed under sterile conditions in the operating theatre. The participants were placed lying on the operating table. Local anesthesia (Lidocaine + Epinephrine) was applied to the abdominal area from which adipose tissue was collected. Approximately 500 mL of saline containing 50 mL of 2% lidocaine and 1 ampoule of Epinephrine was injected to reduce bleeding and tissue trauma. A Carraway Harvester (2.1 mm × 15 cm) connected to the VacLock syringe was then inserted through a small stab incision, and up to 60 mL of adipose tissue was collected into the syringe by pulling the syringe plunger back and forth. A smaller part (4 mL) of obtained lipoaspirate was taken as the first sample source for flow cytometry analysis (LA).

The obtained LA was then divided into several (up to 4) separate syringes (Arthrex ACP^®^ Double-Syringe System, Arthrex GmbH, Munich, Germany) and centrifuged for 4 min at 2500 rpm (Rotofix 32A centrifuge, Swing-out rotor Cat. No. 1624, Hettich). Upon completion of centrifugation, 3 layers within the syringe were distinguished. The lowest layer, the aqueous fraction, was poured out, whereas the highest layer, the layer of broken adipocyte oil, was removed using the Arthrex ACP^®^ Double-Syringe System. The middle layer, a layer of autologous conditioned adipose tissue, was mixed with the same layers of the other syringes through a 1.4 mm wide transfer device at least 30 times to obtain microfragmented lipoaspirate; a small part of this product (2 mL) was set aside as the second sample source for the flow cytometry analysis (MLA).

Most of MLA was centrifuged again for 4 min at 2500 rpm. Again, the oil, which was located in the upper layer, was separated and discarded, and the aqueous fraction was poured out. The middle layer, consisting of concentrated SVF containing mesenchymal MSC cells, was isolated, and a smaller part of it (1 mL) was taken as the third sample for flow cytometry analysis (SVF). The same procedure was repeated for each patient.

The LA, MLA and SVF samples from each patient were kept at room temperature and sent to the Department for Translational Medicine of the Srebrnjak Children’s Hospital, where the flow cytometry analysis was performed.

### 4.3. Cell Isolation

To maximize the cell yield from all three sample types and properly prepare cells for flow cytometry, we equally treated LA, MLA and SVF samples with 1% collagenase type I in Dulbecco’s Modified Eagle Medium (D-MEM) (both from Sigma-Aldrich, Saint Louis, MO, USA) in a shaking bath at 37 °C for 45 min. After 1:2 dilution with 10% heat-inactivated fetal bovine serum (Biosera, Nuaille, France) in D-MEM (Sigma-Aldrich), samples were filtrated through a 100 μm cell strainer (BD Falcon, Corning, NY, USA) and centrifuged at 300× *g* for 10 min at RT. Supernatants were discarded, and the cell pellet was resuspended in 1 mL of the VersaLyse solution (Beckman Coulter, Miami, FL, USA). After 10 min, samples were filtered through a 40 μm-cell strainer (BD Falcon, Corning, NY, USA), centrifuged at 300× *g* for 10 min at RT and the cell pellet resuspended in D-MEM (Sigma-Aldrich). The cells were counted on the Sysmex XT1800 counter (Sysmex, Kobe, Japan), and sample volumes were adjusted to contain 3 × 10^6^ cell/mL.

### 4.4. Flow Cytometry

Cells isolated from LA, MLA and SVF were stained using a DURAClone SC Mesenchymal Tube reagent (Beckman Coulter, Miami, FL, USA). The DURAClone SC Mesenchymal Tube is a polychromatic reagent that allows the identification of MSC subpopulations based on the use of antibodies specific for the cell surface markers: CD31, CD34, CD45, CD73, CD90, CD105 and CD146, labeled with PB, ECD, APC-AF750, PE, FITC, CD45-PC7 and PC5.5 fluorochromes, respectively. To each tube, 100 μL of cell suspensions with 3 × 10^6^ cell/mL and Live/Dead Yellow Fixable Stain (ThermoFisher, Waltham, MA, USA) were added. Samples were gently mixed and incubated for 15 min at RT, protected from light and then treated with 2 mL VersaLyse solution (Beckman Coulter, Miami, FL, USA) for 10 min, RT, protected from light. After centrifugation (RT, 150× *g*), samples were washed with PBS and centrifuged under the same conditions. The supernatant was discarded, and the cell pellet was resuspended in 500 μL of 0.1% paraformaldehyde (Electron Microscopy Sciences, Hatfield, PA, USA) in phosphate-buffered saline (PBS; Sigma-Aldrich). Cell nuclei were stained with the DRAQ5 dye (BioStatus, Shepshed, Leicestershire, UK) for 15 min, RT, protected from light, before acquisition on a Navios flow cytometer (Beckman Coulter, Miami, FL, USA). Flow cytometry data files (FCS) were analyzed using Kaluza software (Beckman Coulter, Miami, FL, USA). More details about the instrument configuration, daily quality control, reagents used and data analysis can be found in Supplementary Materials Table S1.

### 4.5. Statistical Analysis

Statistics were calculated using parametric or non-parametric tests stated under each figure based on the normality calculation (GraphPad Prism 9.2 for Windows; GraphPad Software, Inc., San Diego, CA, USA). A *p*-value < 0.05 was taken as statistically significant.

### 4.6. Ethics Approval

The study was approved by the Ethics Committee of St. Catherine Specialty Hospital (No. 21/3-1).

## 5. Conclusions

This paper expands an insight into the phenotype diversity of MSC originating from adipose tissue. Our future work is to explore how the heterogeneity of the adipose MSC subpopulations and MSC marker expression, together with the patient’s sex-related condition, is associated with specific functions and OA treatment.

## Figures and Tables

**Table 1:** Summarized results of the main stromal vascular fraction immunophenotypes.

Immunophenotype	Lineage Markers	Mesenchymal Stem/Stromal Cell (MSC) Markers
EP	CD45^−^CD31^+^CD34^+^CD146^±^	CD73^±^CD90^±^CD105^±^
Pericytes	CD45^−^CD31^−^CD34^−^CD146^+^	CD73^±^CD90^+^CD105^−^
SA-ASC	CD45^−^CD31^−^CD34^+^CD146^−^	CD73^high^CD90^+^CD105^−^
Leukocytes	CD45^+^CD31^−^CD34^−^CD146^−^	CD73^−^CD90^−^CD105^−^

## Supplementary Materials

The following are available online at https://www.mdpi.com/article/10.3390/genes12121999/s1: Figure S1: Differences between female and male patients in the progenitor cell ratios in stromal vascular fraction from LA, MLA and MLA samples; Table S1: Table S1_MIFlowCyt Item Checklist_IJMS.
