# Supplementary material for: Polychromatic Flow Cytometric Analysis of Stromal Vascular Fraction from Lipoaspirate and Microfragmented Counterparts Reveals Sex-Related Immunophenotype Differences
Source: Genes (Basel). 2021 Dec 16;12(12):1999. doi: 10.3390/genes12121999 (PMC8702056; doi:10.3390/genes12121999)
Supplement: Supplementary file 1 [file genes-12-01999-s001.zip › Supplementary files/Table S1_MIFlowCyt Item Checklist_IJMS.pdf]

genes

MDPI

Author Checklist: MIFlowCyt-Compliant Items

Requirement	Please Include Requested Information
1.1. Purpose	The purpose of the study was to characterize stromal vascular fraction cells from 16 osteoarthritic patients. From each patient two microfragmented lipoaspirate counterparts (named MLA and SVF), of which MLA was applied for cartillage treatment in OA, where compared with autologous lipoaspirate (named LA)
1.2. Keywords	Lipoaspirate, microfragmented lipoaspirate, immunophenotyping, endothelial progenitors, pericytes, supra-adventitial adipose stromal cells, sex.
1.3. Experiment variables	Microfragmented lipoaspirate treatment, gender.
1.4. Organization name and address	Department for Translational Medicine, Srebrnjak Children's Hospital, Srebrnjak 100, Zagreb, Croatia
1.5. Primary contact name and email address	Lucija Zenic, lzenic@bolnica-srebrnjak.hr
1.6. Date or time period of experiment	Data were collected and analysed between March 2021 and June 2021.
1.7. Conclusions	Comparative polychromatic flow cytometry analysis of cells in stromal vascular fraction isolated after collagenase treatment from three type of samples for each patient was performed.<br>The immunophenotyping profile of MLA and SVF was dominated by reduction of leukocytes and augmentation of EP and pericytes, evidencing a marked enrichment of those cell populations in the course of adipose tissue microfragmentation. The increase of pericytes and EP as well as the decrease of leukocytes did not differ be-tween MLA and SVF samples indicating that the method of lipoaspirate microfragmentation involving one centrifugation step was equally efficacious in the enrichment of the re-generative cell compartment as the method of concentrating stromal vascular fraction in-volving two centrifugation steps<br>The pericytes/SA-ASC and EP/SA-ASC ratios were significantly higher in MLA and SVF samples compared to LA samples. The same ratios showed statistical difference when the samples from female and male patients were compared, however, statistically higher ratios in men were only seen in LA but not in MLA and SVF samples.<br>Differential expression of the CD31 and CD34 markers reflecting two discerned sub-populations of endothelial progenitor cells was detected: the more abundant $CD31^{+}CD34^{+}$ EP and the less abundant $CD31^{high}CD34^{high}$. The proportion of the $CD31^{high}CD34^{high}$ EP was significantly higher while the proportion of $CD31^{+}CD34^{+}$ EP was statistically lower in MLA and SVF compared to LA. Furthermore, the level of the CD31 and CD34 expression differed based on sex, and in female patients the proportion of $CD31^{high}CD34^{high}$ EP was statistically

	higher while the proportion of CD31$^+$CD34$^+$ EP was statistically lower compared to male patients in all LA, MLA and SVF samples.<br>With respect to the MSC marker expression significantly higher expression of the CD90, CD105 and CD146 markers was determined on CD31^{high}CD34^{high} EP in LA from female patients compared to their male counterparts. The same CD31^{high}CD34^{high} EP subpopulation showed significantly higher expression of CD105 in MLA samples and CD146 in SVF samples from female patients compared to male patients.<br>When the same data were compared for CD31$^+$CD34$^+$ EP and CD31^{high}CD34^{high} EP within each sex, statistically higher expression of the CD90, CD105 and CD146 markers was determined on CD31^{high}CD34^{high} EP in both female and male patients compared to its expression on CD31$^+$CD34$^+$ from all LA, MLA and SVF samples.<br>Analysis of MSC markers and the CD146 adhesion molecule expression in other progenitor populations revealed that female patients showed significantly higher expression of CD90 on pericytes and CD73 and CD90 on SA-ASC from all LA, MLA and SVF samples.
1.8. Quality control measures	FlowCheck-pro beads and Flow-Set Pro beads were used daily for QC and to monitor sensitivity. VersaComp beads was used for the compensation. All beads are from Beckman Coulter.
2.1.1.1. (2.1.2.1., 2.1.3.1.) Sample description	1. LA = Human lipoaspirate<br>2. MLA = Human microfragmented lipoaspirate (from LA, 1st time centrifuged using ACP® Double-Syringe System)<br>3. SVF = Human microfragmented lipoaspirate (from MLA, 2nd time centrifuged using ACP® Double-Syringe System.)
2.1.1.2. Biological sample source description	LA, MLA and SVF samples were obtained from sixteen patients with osteoarthritis (8 males and 8 females, aged 33-66). The LA, MLA and SVF patient samples were delivered to the Srebrnjak Children's Hospital (Zagreb, Croatia) and immediately processed.
2.1.1.3. Biological sample source organism description	Homo sapiens, 8 females, 8 males.
2.1.2.2. Environmental sample location	N/A
2.1.3. Other samples	Flow-Check Pro Fluorospheres (Beckman Coulter A63493)<br>Flow-Set pro Fluorospheres (Beckman Coulter A63492)<br>BD Anti-Mouse Ig, κ/Negative Control Compensation Particles Set (BD 552843)
2.3. Sample treatment description	LA, MLA and SVF samples were digested with 1 % collagenase type I in D- MEM (both from Sigma-Aldrich, Saint Louis, MO, USA) in a shaking bath at 37°C for 45 minutes. After dilution 1:2 with 2% heat-inactivated fetal bovine serum (Biosera, Nuaille, France) in D-MEM (Sigma-Aldrich), samples were filtrated through a 100 μm-cell strainer (BD Falcon, Corning, NY) and washed with D-MEM (Sigma-Aldrich). Supernatants were discarded and cell pellets resuspended in 1 ml of D-MEM (Sigma-Aldrich). Samples were filtered through a 40 μm-cell strainer (BD Falcon, Corning, NY), washed with D-MEM (Sigma-Aldrich) and cell pellets resuspended in 1 ml of D-MEM D-MEM (Sigma-

	Aldrich). Aliquote of 200µl from each sample was taken, cells counted on the Sysmex XT1800 counter (Sysmex, Kobe, Japan) and for each sample cell concentration adjusted to 3 x 106 cell/ml.
2.4. Fluorescence reagent(s) description	

INSTRUMENT		ANTIBODIES / DYES				
LASER	NAVIOS DETECTOR	TARGET	Clone	FLUOROCHROME / DYE	CATALOG #	MANUFACTURER
488nm	FL1	CD90	F15_42	FITC	DURAClone SC Mesenchymal Tube reagent	Beckman Coulter
	FL2	CD73	AD2	PE	DURAClone SC Mesenchymal Tube reagent	Beckman Coulter
	FL3	CD34	581	ECD	DURAClone SC Mesenchymal Tube reagent	Beckman Coulter
	FL4	CD146	TEA1/34	PC5.5	DURAClone SC Mesenchymal Tube reagent	Beckman Coulter
	FL5	CD105	TEA3_17	PC7	DURAClone SC Mesenchymal Tube reagent	Beckman Coulter
638 nm	FL6	/	/	/	/	/
	FL7	DNA	/	DRAQ5	DR50200	BioStatus
	FL8	CD45	J33	AA750	DURAClone SC Mesenchymal Tube reagent	Beckman Coulter
405 nm	FL9	CD31	5_6E	Pacific Blue	DURAClone SC Mesenchymal Tube reagent	c
	FL10	CD14, CD19, Dead cells	RM052, J3-119, Live/Dead Fixable Yellow	Krome Orange, Yellow	DURAClone SC Mesenchymal Tube reagent + Live/Dead Fixable Yellow	Beckman Coulter , ThermoFisher

3.1. Instrument manufacturer	Beckman Coulter
3.2. Instrument model	Navios
3.3. Instrument configuration and settings	The flow cytometer Navios has been configured by the manufacturer. For the set up of the instrument "EuroFlow SOP for Instrument set-up and compensation for Navios Instruments Version 1.4 was used (https://www.euroflow.org/usr/pub/protocols.php).
3.3.1. Flow Cell and Fluidics	Sensing area: BioSense 150 µm x 460 µm rectangular cuvette. Flow-Check Pro and Flow-Set Pro beads were acquired at approximately 10 µl/min. Compensation beads, LA and MLA samples were acquired at approximately 60 µL/min.
3.3.2. Light sources	Navios is equipped with three solid-state, software controlled lasers.

	1) 22 mW, blue laser operating at 488 nm, elliptical beam spot 10 μm x 84 μm<br>2) 25 mW, diode laser operating at 638 nm, elliptical beam spot 9.6 μm x 72 μm<br>3) 40 mW, violet laser operating at 405 nm, elliptical beam spot 8.9 μm x 70 μm
3.3.3. Optical fliters	Optical configuration and transmitted wavelengths are show at diagram bellow (diagram was taken from "Navios Flow Cytometer: Instructions for Use" manual that was supplied by Beckman Coulter at the time of instrument pucrchase during the year 2012.

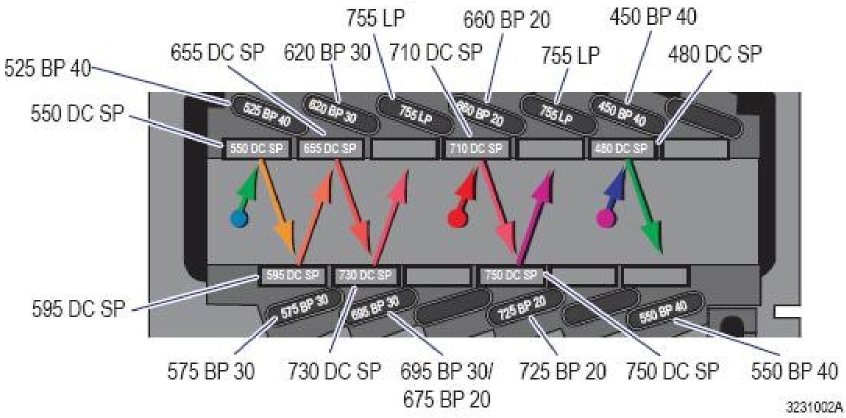

3.3.4. Optical detectors	For 488 nm laser: FL1 – FL5<br>For 638 nm laser: FL6 – FL8<br>For 405 laser: FL9 - FL10
3.3.4.1. Optical detector types, voltages, amplification	Data about detector and instrument settings are listed in table below:

Laser	Detector	Detector type	Parameter / Fluorochrome	Target channel value (MFI) for Flow-Set Pro Beads	Voltage settings	Amplification
2488 nm	Wide	Segmented photodiode	FSC Intensity and FSC Time of Flight	/		Linear
	/	Photodiode	SSC Intensity and SSC Time of Flight	/		Linear
	FL1	PMT	FITC	68-76	500	Log
	FL2	PMT	PE	232-262	500	Log
	FL3	PMT	ECD	146-166	425	Log
	FL4	PMT	PC5..5	116-124	550	Log
	FL5	PMT	PC7	54-58	570	Log
638 nm	FL6	PMT	APC	15-17	485	Log

	FL7	PMT	DRAQ7	260-296	450	Log
	FL8	PMT	AA750	73-83	485	Log
405 nm	FL9	PMT	Pacific Blue	55-61	425	Log
	FL10	PMT	Live/Dead Yelow	110-124	348	Log

4.2. Compensation description	Tubes with single dried DuraClone reagent were used for compensation samples as follow: CD4 FITC, CD4 PE, CD34 ECD, CD146 PE-Cy5.5, CD105 PE-Cy7, CD45 APC-AlexaFluor750 and CD8 Pacific Blue. VersaComp beads were added to each tube. Then 100 µl of PBS was added, tubes briefly vortexed and proceeded further in the same manner as the DuraClone SC tubes. For the DRAQ5, compensation mixture of DRAQ5 stained and unstained SVF cells was used. Compensation was not performed for the Live/Dead Fixable Yellow stain because only negative cells were used in analysis of MSCs.
4.3. Data transformation details	N/A
4.4.1. Gate description	Gating procedures applied was, with minor modifications, as described in article:<br>Polancec, D.; Zenic, L.; Hudetz, D.; Boric, I.; Jelec, Z.; Rod, E.; Vrdoljak, T.; Skelin, A.; Plecko, M.; Turkalj, M.; Nogalo, B.; Primorac, D. Immunophenotyping of a Stromal Vascular Fraction from Microfragmented Lipoaspirate Used in Osteoarthritis Cartilage Treatment and Its Lipoaspirate Counterpart. Genes 2019, 10, 474.<br><br>The gating tree was set as follows (as shown on Figures :<br>1. FSC LIN/FSC TOF: „Singlets“: exclusion of doublets, to<br>2. SSC/DRAQ5: „Nucleated cells“: exclusion of debris and selection of nucleated cells, to<br>3. DRAQ5/CD45 APC-AlexaFluor750: Selection of CD45$^-$ cells and CD45+ cells<br>4. CD45$^-$ cells were further analysed on CD34 ECD / CD31 Pacific Blue for the determination of CD31$^+$CD34$^-$ endothelial mature cells, determination of CD31$^+$CD34$^+$ endothelial progenitor cells and selection of CD31$^-$ cells for further analysis<br>5. CD31$^-$ cells were further analysed for CD34 ECD/CD146 PC5.5for the determination of CD34$^-$CD146$^+$ pericytes and CD34$^+$CD146$^-$ supra-adventitial adipose stromal cells.<br>6. CD31$^+$CD34$^+$ endothelial progenitor cells were divided into CD31^{high}CD34^{high} endothelial progenitors and CD31$^+$CD34$^+$ endothelial progenitors
4.4.2. Gate statistics	Percentage positive from nucleated events were determined for CD45+ cells and percentage positive from live nucleated CD45- events for endothelial progenitor cells, pericytes and SA-ASC.

	Percentage positive from endothelial progenitors were determined for CD31^{high}CD34^{high} and CD31^{+}CD34^{+} endothelial progenitors.<br>Geometric mean fluorescence intenstiy was determind for mesenchymal stem/stromal cell-characteristic surface markers CD73, CD90, CD105 as well as for CD146 cell surface marker, on endothelial progenitors, pericytes and supra-adventitial adipose stromal cells.
4.4.3. Gate boundaries	Regions and gates were positioned according to gating strategies published in article listed under the 4.4.1. section. Batch processing of the FCS data analisys was performed for all eighteen samples and regions and gates minimaly adjusted were needed.
