# Supplementary figures and images for: Polychromatic Flow Cytometric Analysis of Stromal Vascular Fraction from Lipoaspirate and Microfragmented Counterparts Reveals Sex-Related Immunophenotype Differences
Source: Genes (Basel). 2021 Dec 16;12(12):1999. doi: 10.3390/genes12121999 (PMC8702056; doi:10.3390/genes12121999)

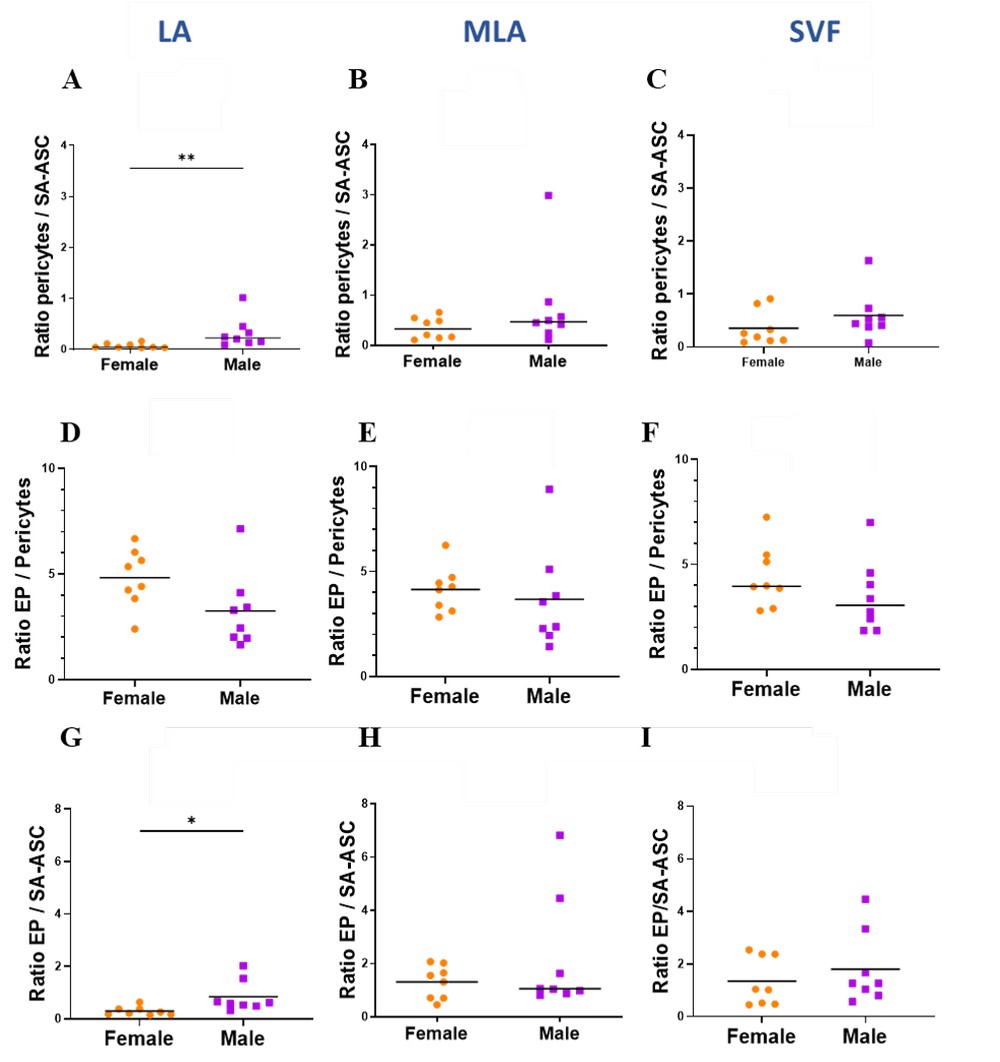

Supplement: Supplementary file 1 [file genes-12-01999-s001.zip › Supplementary files/Figure S1.jpg]
